# An MRI-Visible Non-Viral Vector Bearing GD2 Single Chain Antibody for Targeted Gene Delivery to Human Bone Marrow Mesenchymal Stem Cells

**DOI:** 10.1371/journal.pone.0076612

**Published:** 2013-10-07

**Authors:** Pengfei Pang, Chun Wu, Min Shen, Faming Gong, Kangshun Zhu, Zaibo Jiang, Shouhai Guan, Hong Shan, Xintao Shuai

**Affiliations:** 1 Molecular Imaging Lab, The Third Affiliated Hospital of Sun Yat-sen University, Guangzhou, China; 2 PCFM Lab of Ministry of Education, School of Chemistry and Chemical Engineering, Sun Yat-sen University, Guangzhou, China; 3 Interventional Radiology Institute of Sun Yat-sen University, Guangzhou, China; 4 Department of Radiology, the Third Affiliated Hospital of Sun Yat-sen University, Guangzhou, China; Argonne National Laboratory, United States of America

## Abstract

The neural ganglioside GD2 has recently been reported to be a novel surface marker that is only expressed on human bone marrow mesenchymal stem cells within normal marrow. In this study, an MRI-visible, targeted, non-viral vector for effective gene delivery to human bone marrow mesenchymal stem cells was first synthesized by attaching a targeting ligand, the GD2 single chain antibody (scAb_GD2_), to the distal ends of PEG-g-PEI-SPION. The targeted vector was then used to condense plasmid DNA to form nanoparticles showing stable small size, low cytotoxicity, and good biocompatibility. Based on a reporter gene assay, the transfection efficiency of targeting complex reached the highest value at 59.6% ± 4.5% in human bone marrow mesenchymal stem cells, which was higher than those obtained using nontargeting complex and lipofectamine/pDNA (17.7% ± 2.9% and 34.9% ± 3.6%, respectively) (*P*<0.01). Consequently, compared with the nontargeting group, more in vivo gene expression was observed in the fibrotic rat livers of the targeting group. Furthermore, the targeting capacity of scAb_GD2_-PEG-g-PEI-SPION was successfully verified in vitro by confocal laser scanning microscopy, Prussian blue staining, and magnetic resonance imaging. Our results indicate that scAb_GD2_-PEG-g-PEI-SPION is a promising MRI-visible non-viral vector for targeted gene delivery to human bone marrow mesenchymal stem cells.

## Introduction

Human mesenchymal stem cells (hMSCs) have the capability for self-renewal and differentiation into a broad spectrum of mesenchymal tissues, such as bone, fat, and cartilage [1-3]. To date, human bone marrow mesenchymal stem cells (hBMSCs) have been widely studied because they are considerably more abundant and easier to obtain compared with hMSCs existing in the stroma of other tissues [4,5]. Mesenchymal stem cell transplantation has become a promising treatment for many diseases such as diabetes, liver fibrosis, and myocardial infarction [6-8]. However, in contrast to hematopoietic stem cells, there has been a lack of definitive cell markers to uniquely identify hBMSCs in their primitive state. Recently the neural ganglioside GD2 has been reported to be a novel surface marker that is only expressed on hBMSCs within normal marrow [9]. More importantly, GD2 expression was at a high level and the entire population of expanded hBMSCs maintained similarly high levels of GD2 expression through 8 culture passages. Furthermore, hMSCs derived from umbilical cord were also reported to be the only cells that expressed this marker within umbilical cord at early-passages [10].

To obtain gene-modified stem cells that possess superior characteristics [11,12], different viral gene delivery vectors, including retroviruses, adenoviruses, and adeno-associated viruses, have been extensively used to deliver ectogenic genes to stem cells [13,14]. Although satisfactory transfection efficiency has been achieved, the potential life-threatening effects of immunogenicity and carcinogenicity restrict further application of them to humans. With the development of nanomedicines, non-viral gene vectors have become alternative, safe, and efficient gene delivery systems because of their stability, safety, ease in preparation, and lack of immunogenicity [15-17]. Polyethyleneimine (PEI), a branched polyamine, was reported to be one of the most commonly used non-viral gene carriers [18]. However, high cytotoxicity and interaction with serum protein are still the major limiting factors for PEI-mediated gene delivery [19]. Flow cytometry revealed that the highest transfection efficiency using PEI as a carrier for gene delivery to human adipose tissue derived stem cells was only 19% [20]. Therefore, polyethylene glycol (PEG) was introduced to PEI (PEG-g-PEI) to reduce cytotoxicity. However, PEGylation of PEI also resulted in a major drawback. That is, the PEG layer shielding reduces the interaction between the nanocomplexes and cells, which essentially lowers the gene transfection efficiency. In this case, targeting ligand modification of vector may enhance the interaction between the nanocomplexes and cells for ideal transfection efficiency, while the advantages of PEGylation are reserved. Chen et al [21] reported that targeting ligand functionalization of PEG-g-PEI leads to 16 fold enhancement in the gene transfection level in rat T lymphocyte cells. In our previous studies [22], we had constructed an MRI-visible targeted siRNA delivery system (scAb_GD2_-PEG-g-PEI-SPION) by complexing PEG-g-PEI with superparamagnetic iron oxide nanoparticles (SPION) and attaching a targeting ligand scAb_GD2_ to the distal ends of PEG-g-PEI-SPION.

**Figure 1:**
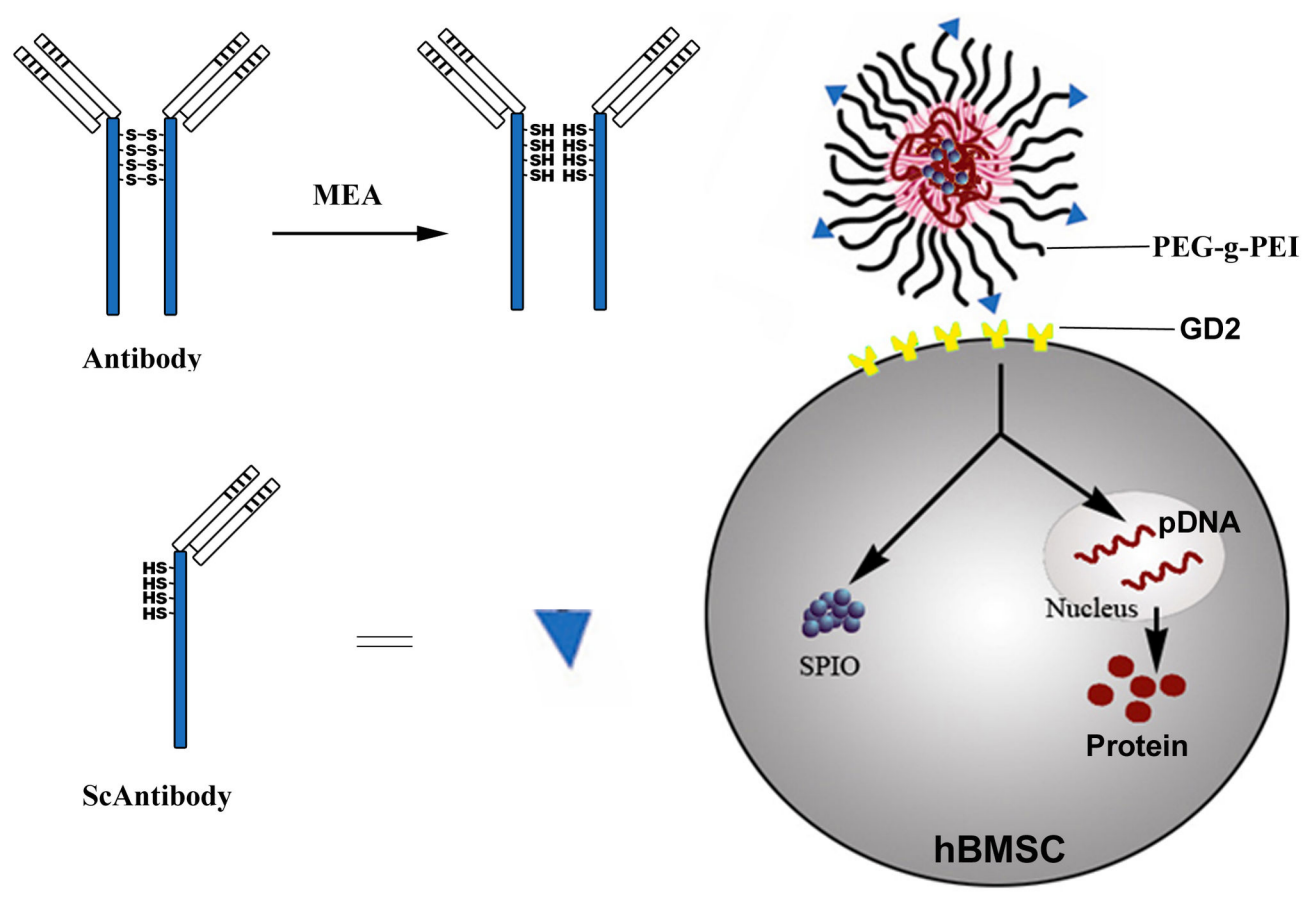
Schematic diagram of targeted gene delivery to hBMSCs.

Based on these achievements, we speculate that GD2 receptors may also mediate potentially targeted gene delivery to hBMSCs (Figure 1). In this study, we prove that scAb_GD2_-PEG-g-PEI-SPION is an efficient MRI-visible non-viral vector for targeted gene delivery to hBMSCs in vitro and in vivo.

## Materials and Methods

### Materials

PEG-g-PEI-SPION was synthesized by our labs as previously reported [22]. Dulbecco’s modified Eagle’s medium (DMEM, low glucose), fetal bovine serum (FBS) and Cell Counting Kit-8 (CCK-8) were purchased from Invitrogen Corporation (Carlsbad, CA, USA). Adipogenic and osteogenic media were purchased from Cyagen Biosciences, Inc. (Guangzhou, China). Fluorescent staining agents 4′, 6-diamidino-2-phenylindole (DAPI), popo-3, and Oregon Green 488 carboxylic acid, succinimidyl ester were obtained from Molecular Probes, Inc. (Eugene, OR, USA). Lipofectamine™ 2000 was used as a reference and was purchased from Beyotime Institute of Biotechnology (Guangzhou, China). The plasmid expressing enhanced green fluorescent protein (pEGFP-C1, 4.7 kb) was expanded in *Escherichia coli* (*E. coli* strain DH5α) for 16 h and then purified by EndoFree Plasmid Giga Kits (QIAGEN, CA, USA) according to the manufacturer’s protocol. The quantity and quality of the purified plasmid DNAs (pDNAs) were determined by optical density at 260 and 280 nm and by electrophoresis in 1% agarose gel. The purified pDNAs were resuspended in deionized water and kept in aliquots at a concentration of 2.4 µg/µL. hBMSCs isolation for research was approved by the institutional review board of the third affiliated hospital of Sun Yat-Sen University according to a protocol previously described [23]. The mouse anti-human GD2 monoclonal antibody (14.G2a), isotype antibody mouse IgG2a, and rabbit monoclonal antibody against EGFP were purchased from BD Bioscience Pharmingen (San Jose, CA, USA).

### hBMSCs isolation and characterization

hBMSCs were harvested from multiple randomized healthy volunteers with informed consent. There were 5 males and 3 females with a mean age of 27.1 ± 4.6 years (range, 21-35 years). Briefly, 10 mL of bone marrow was aspirated from the iliac crest of each volunteer. hBMSCs were isolated from each sample by loading onto Percoll solution (d=1.077 g/mL). After centrifugation at 900 g for 25 min, the MSC layer was removed from the interphase and washed 3 times with phosphate buffered saline (PBS). Then, the cells were resuspended in culture medium (DMEM supplemented with 10% FBS, 100 U/mL penicillin, and 100 µg/mL streptomycin) and planted into a 25 cm^2^ tissue culture flask at a concentration of 1×10^6^ cells/cm^2^. The culture medium was replaced to remove nonadherent cells after 72 h and changed every 3-4 days throughout the studies. For characterization, hBMSCs were incubated with fluorescent-conjugated antibodies (1 μg/10^6^ cells) for CD73, CD105, CD34, and CD45 (BD PharMingen, San Jose, CA, USA) at 4°C in the dark for 30 min and then washed with PBS. Fluorescent-conjugated isotype-matched control IgG1 was used to evaluate nonspecific background. The cells were analyzed using a FACScan flow cytometer (Becton Dickinson, Franklin Lakes, NJ, USA). For GD2-positive cells isolation, hBMSCs (P2) were incubated with mouse anti-human GD2 monoclonal antibody (2 μg/10^6^ cells) at 4°C for 30 min. The cells were then incubated with goat anti-mouse IgG microbeads according to the manufacturer’s protocol.GD2-positive cells were obtained by using a magnetic separation column (Miltenyi Biotec). The final hBMSCs used were taken at passage 3-5.

### Synthesis of scAb_GD2_-PEG-g-PEI-SPION

In brief, 200 µL of GD2 antibody (0.5mg/mL) was mixed with 200 µL of ethylenediaminetetraacetic acid (EDTA) solution (0.5 M, pH=8.0). 100 mg of 2-mercaptoethylamine (MEA) and 20 µL of 0.5 M EDTA solution were dissolved in PBS (500 µL) and then were mixed with the antibody solution. After incubation for 90 min at 37°C, the obtained scAb_GD2_ solution was washed 3 times with PBS (pH7.4, each 500 µL containing 10 µL of 0.5 M EDTA solution) using an Amicon cell (MWCO=10 kDa) to remove the excess MEA. 200 µg of Mal-PEG-COOH dissolved in 200 µL of PBS (pH7.4, each 500 µL containing 10 µL of 0.5 M EDTA solution) was added into the scAb_GD2_ solution and then incubated at 4°C overnight. The resultant scAb_GD2_-functionalized PEG (scAb_GD2_-PEG-COOH) solution was washed 3 times with fresh PBS (pH7.4) using an Amicon cell (MWCO=10 kDa). 10 µg of both 1-ethyl-3-(3-dimethylaminepropyl) carbodiimide (EDC) and N-hydroxysuccinimide (NHS) were added into the purified solution and incubated at 4°C for 10 min. 200 µg of PEG-g-PEI-SPION was then added, and the solution was incubated overnight at 4°C to obtain scAb_GD2_-PEG-g-PEI-SPION.

### Complex formation

The plasmid DNA (1µg) and an appropriate amount of the delivery agents (PEG-g-PEI-SPION, scAb_GD2_-PEG-g-PEI-SPION) in accord with the desired N/P ratio (molar ratio of the positive amino groups of delivery agents to the phosphoric anions of plasmid DNA) were separately diluted with ultrapure water. The two solutions were fully mixed by vigorous pipetting and then were kept at room temperature for 30 min to allow complex formation.

### Particle size and zeta potential measurements

Complexes (PEG-g-PEI-SPION/pDNA, scAb_GD2_-PEG-g-PEI-SPION/pDNA) were prepared at designated N/P ratios of 5, 10, 15, 20, 25, 30, 35, and 40. The particle size and zeta potential of the complexes (1mL) were measured by dynamic light scattering (DLS, ELS-8000, Photal, Japan) at room temperature.

### Agarose gel retardation assay

Gel electrophoresis was performed to assess the pDNA condensation ability of the delivery agents on a Bio-Rad Sub-Cell electrophoresis cell (Bio-Rad Laboratories, Inc, USA). Complexes (PEG-g-PEI-SPION/pDNA, scAb_GD2_-PEG-g-PEI-SPION/pDNA) were prepared at various N/P ratios from 2.1 to 2.5 as already described. The complexes were mixed with 50% glycerin, loaded into 1% agarose gel with ethidium bromide (0.5 µg/mL) and then were run with Tris-acetate (TAE) buffer at 120 V for 25 min. The gel images were captured on a DNR Bio-Imaging System (DNR Bio-Imaging Systems Ltd, Israel).

### In vitro cytotoxicity assay

hBMSCs were seeded at a density of 8000 cells/well on 96-well plates in 100 µL of complete DMEM and cultured for 24 h at 37°C in a fully humidified atmosphere of 5% CO_2_. The cells were then incubated with complexes (PEG-g-PEI-SPION/pDNA, scAb_GD2_-PEG-g-PEI-SPION/pDNA) formed at various N/P ratios for another 24 h. The pDNA mass in each well was set to 0.15 µg. After 10 µL of CCK-8 solution was added into each well, the cells were incubated for an additional 3 h. The absorbance at 450 nm was recorded on a Tecan Infinite F200 Multimode plate reader. All experiments were conducted in triplicate.

### In vitro gene transfection

hBMSCs were seeded at a density of 2×10^5^ cells/well on 6-well plates and cultured for 12 h before transfection. The Complexes (PEG-g-PEI-SPION/pDNA, scAb_GD2_-PEG-g-PEI-SPION/pDNA) prepared at designated N/P ratios of 0, 10, 15, 20, 30, and 40 were added into the culture medium. A lipofectamine/pDNA complex prepared with 10 µL of lipofectamine was used as reference. The pDNA mass in each well was set to 4 µg. hBMSCs were incubated for 12 h with each complex. The hBMSCs were then washed with PBS, complete DMEM was added, and the cells were further cultured for 40 h. The expression of EGFP was observed under a Carl Zeiss Aviox-1 inverted fluorescence microscope. To evaluate transfection efficiency, the cells were harvested and resuspended in cold PBS. The transfection efficiency was determined using a FACScan flow cytometer (Becton Dickinson, Franklin Lakes, NJ, USA). The fluorescence parameters were acquired using at least 10,000 events per sample. All transfection experiments were performed in triplicate. Data analysis was performed by Becton Dickinson CellQuest Software.

The free antibody competitive inhibition assay and isotype antibody (mouse IgG2a) assay were performed to confirm the specificity of scAb_GD2_-PEG-g-PEI-SPION. For free antibody competitive inhibition assay, cells were incubated with a large amount of free GD2 antibody for 30 min before complex scAb_GD2_-PEG-g-PEI-SPION/pDNA was added into the culture medium. Isotype antibody assay was performed by attaching scAb_IgG2a_ to the distal ends of PEG-g-PEI-SPION. hBMSCs were then incubated with complex scAb_IgG2a_-PEG-g-PEI-SPION/pDNA for 12 h. The level of gene expression was evaluated as already described.

### hBMSCs differentiation and transwell migration assays

The multilineage differentiation potential of hBMSCs was confirmed by testing their ability to differentiate into adipocytes and osteoblasts. Briefly, hBMSCs were seeded on 6-well plates and incubated with complexes formed at an N/P ratio of 20 for 12 h. Then, the cells were further cultured in adipogenic and osteogenic media for approximately 3 weeks. Adipocyte and osteoblast differentiation was identified by Oil red O and Alizarin red S, respectively.

Transwell migration assay was performed to evaluate the effect of the complexes on the migration ability of hBMSCs. Briefly, hBMSCs were seeded on 6-well plates and incubated with complexes (scAb_GD2_-PEG-g-PEI-SPION/pDNA, PEG-g-PEI-SPION/pDNA) formed at an N/P ratio of 20 for 12 h. Then, the cells were harvested and washed 3 times with PBS. A total of 2×10^4^ cells in 200 µL of serum-free DMEM were added into the upper chamber of a 24 well transwell plate (8 µm, Corning Costar, New York, USA). The lower chamber was filled with DMEM supplemented with 10% FBS. After incubation at 37°C for 12 h, hBMSCs penetrated through the permeable membrane were fixed with 4% paraformaldehyde for 15 min and stained with Giemsa for 10 min. The upper surface of the permeable membrane was carefully wiped with a cotton swab. hBMSCs penetrated through the permeable membrane were counted in five non-overlapping high power field (HPF) and photographed. Normal hBMSCs were used as control. All transwell experiments were performed in triplicate.

### Cellular uptake of complexes

To study the cellular uptake of the complexes, confocal laser scanning microscopy (CLSM) experiment was performed. In brief, Oregon Green 488 and a delivery agent, such as scAb_GD2_-PEG-g-PEI-SPION, were dissolved in dimethyl sulfoxide (1 mg/mL) and sodium bicarbonate buffer (0.1 M, pH 8.3-9.0), respectively. The Oregon Green 488 solution was slowly added into the delivery agent solution under stirring. The mixture was stirred in the dark for 1 h at room temperature. The conjugate solution was washed several times with PBS (pH 7.4) using ultrafiltration in an Amicon cell (regenerated cellulose membrane, MWCO=5 kDa) until no absorption at 488 nm was detectable in the filtrate. The pDNA was labeled with popo-3 nucleic acid stain (1mg/mL in dimethyl sulfoxide, Molecular Probes) according to the manufacturer’s protocol. Similarly, the popo-3 solution was slowly added into the pDNA solution, and the mixture was also stirred in the dark for 1 h at room temperature. Gel filtration (Sephadex G-25, GE Healthcare UK Limited, Buckinghamshire, UK) was used to purify the pDNA-popo-3 conjugate solution.

hBMSCs were seeded on 35 mm sterile glass-bottom culture dishes (MatTeK, USA) with complete DMEM. The popo-3-labeled pDNA (4 µg) was mixed with the appropriate amount of Oregon Green488-labeled PEG-g-PEI-SPION/ scAb_GD2_-PEG-g-PEI-SPION (N/P ratio of 20). The cells were cultured for an additional 6 h in the presence of the prepared complexes and washed 3 times with fresh PBS to remove free complexes. After being fixed in a 4% paraformaldehyde solution for 10 min, the cells were further incubated for another 15 min with the DNA-staining agent DAPI (1 mg/mL) and washed 3 times with fresh PBS. CLSM images were acquired using a Zeiss LSM 510 META microscope (Carl Zeiss Meditec, Göttingen, Germany). The free antibody competitive inhibition assay was also performed.

### Prussian blue staining

Prussian blue staining was performed to confirm the presence of iron particles (SPION) in the cytoplasm of the cells. After being incubated with complexes formed at an N/P ratio of 20 for 6 h, the cells were washed 3 times with fresh PBS, fixed with 4% glutaraldehyde for 10 min, washed again, and then incubated with 2 mL of Prussian blue solution containing 1% hydrochloride and 1% potassium ferrocyanide (II) trihydrate for 30 min. A Zeiss microscope was used to evaluate the iron-staining effect.

### In vitro MRI scan

hBMSCs were seeded at a density of 2 × 10^5^ cells/well on 6-well plates, incubated for 6 h in the presence of complexes-containing SPION at Fe concentrations of 0, 10, 20, 40, 60 µg/mL in complete DMEM, washed 3 times with PBS, resuspended in a 4% gelatin solution, and then scanned with a 1.5 T MR scanner (GE Healthcare UK Limited, Buckinghamshire, UK) using a 3-inch surface coil at room temperature. T_2_-weighted images were acquired using the following parameters: TR/TE, 2000/100 ms; FOV, 200 ×200 mm; matrix, 384 ×256; slice thickness, 1.0 mm. The imaging parameters for T_2_*-weighted images were TR/TE, 400/15 ms; FOV, 200 ×200 mm; matrix, 384 ×256; flip angle, 15°; slice thickness, 1.0 mm. A circular 30 mm^2^ region of interest was used to determine the signal intensity of the cells. The signal intensity of the treated cells was normalized by comparing it with the intensity of blank cells. 

### In vivo gene expression

hBMSCs transfected with scAb_GD2_-PEG-g-PEI-SPION/PEG-g-PEI-SPION were transplanted into fibrotic rat livers to confirm gene expression in vivo. Liver fibrosis was induced by carbon tetrachloride (CCL_4_) as we previously reported [24]. The animal use protocol was approved by the Institutional Animal Care and Use Committee of Sun Yat-Sen University. Briefly, 0.033 mL of CCL_4_ per 100 g body weight was intraperitoneally administered twice a week to 15 male Sprague-Dawley rats weighing 150-160 g. Liver fibrosis was proved by histological analysis after 10 weeks. Twelve of the rats were randomly divided into targeting group and nontargeting group (n=6 for each group). hBMSCs were incubated with complexes (PEG-g-PEI-SPION/pDNA, scAb_GD2_-PEG-g-PEI-SPION/pDNA) formed at an N/P ratio of 20 for 12 h. After being anesthetized, the rats underwent an abdominal incision to expose the mesenteric vein, into which approximately 5×10^6^ hBMSCs in 1 mL of PBS were slowly administered. The expression of EGFP in the rat livers was detected by fluorescence observation and immunohistochemistry.

For fluorescence observation of EGFP, frozen sections were prepared at the third day post transplantation. Briefly, after rats were perfused with 100 mL of 1.5% paraformaldehyde, liver tissues were dissected, immersed in 20% sucrose solution for 2 h, mounted in OCT (opti-mum cutting temperature compound), and then sectioned using a cryostat. Frozen sections thus prepared were then stained with a solution of 2 µg/mL Hoechst 33342 for 15 min to mark the nucleus. Images were recorded using a Zeiss LSM 510 META microscope (Carl Zeiss Meditec, Göttingen, Germany). EGFP and Hoechst 33342 were excited at 490 nm and 352 nm, respectively.

Immunohistochemistry was also performed to confirm the EGFP-positive cells. The liver tissues were collected at the third day post transplantation, fixed in 4% paraformaldehyde, embedded in paraffin, and then sectioned with a microtome (5 µm thick). The deparaffinized sections were incubated with 3% hydrogen peroxide, blocked with protein blocking serum for 5 min, and then incubated with rabbit monoclonal antibody against EGFP (BD PharMingen, San Jose, CA, USA) at 4°C overnight. After being washed with PBS, the sections were incubated with HRP-conjugated goat anti-rabbit antibody for 30 min and were subsequently stained with 3, 3-diaminobenzidine (DAB). They were then washed with PBS and stained with hematoxylin.

### Statistical analysis

Statistical analysis of the data was performed with one-factor analysis of variance and t-test (SPSS software, version 13.0, SPSS Inc, USA). The results were expressed as the mean ± standard deviation, and a *P* < 0.05 was considered to be statistically significant. All statistical tests were two-sided.

## Results and Discussion

### hBMSCs isolation and characterization

After hBMSCs were isolated from bone marrow and cultured as a monolayer, phase contrast microscopy revealed that most of the adherent cells exhibited a fibroblast-like spindle shape. The hBMSCs proliferated quickly and formed a uniform confluent monolayer in complete DMEM. Flow cytometry was performed to examine the expression of the cell surface antigens CD73, CD105, CD34, and CD45. hBMSCs showed positive expression of the mesenchymal stem cell markers CD73 and CD105, negative expression of the hematopoietic stem cell markers CD34 and CD45 (data not shown).

### Particle size and zeta potential measurements

**Figure 2:**
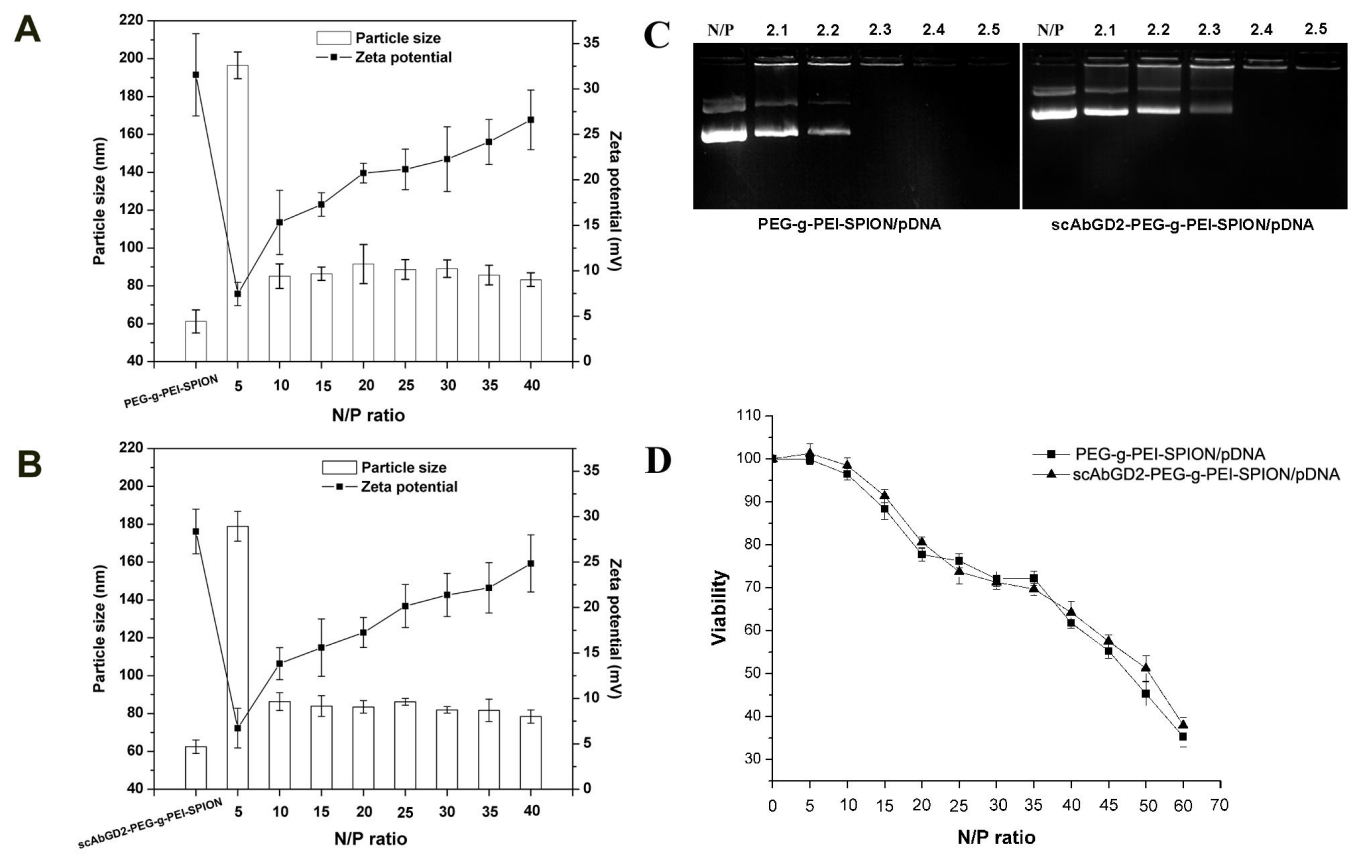
Characterization of the complexes. A, B) Particle size and zeta potential of complexes PEG-g-PEI-SPION/pDNA and scAb_GD2_-PEG-g-PEI-SPION/pDNA. Both PEG-g-PEI-SPION and scAb_GD2_-PEG-g-PEI-SPION condensed pDNA to form stable nanoparticles 80-100 nm in diameter at an N/P ratio of 20. The surface charge of the complexes increased with increasing N/P ratio. C) Electrophoretic migration of complexes scAb_GD2_-PEG-g-PEI-SPION/pDNA and PEG-g-PEI-SPION/pDNA at various N/P ratios. PEG-g-PEI-SPION and scAb_GD2_-PEG-g-PEI-SPION completely bound pDNA at N/P ratios of 2.3 and 2.4, respectively. D) In vitro cytotoxicity of complexes in hBMSCs determined by CCK-8 assay. The CCK-8 assay was used to evaluate the cytotoxicities of the complexes PEG-g-PEI-SPION/pDNA and scAb_GD2_-PEG-g-PEI-SPION/pDNA in hBMSCs. The complexes were formed at various N/P values, and the pDNA mass in each well was set to 0.15 µg. No statistical difference was found between the two groups (*P*>0.05). The results were presented as the mean ± standard deviation (n=3).

The condensation of pDNA into nanoparticles is an important prerequisite for gene delivery using cationic polymers [25]. In this study, the particle size of targeting (scAb_GD2_-PEG-g-PEI-SPION/pDNA) and nontargeting (PEG-g-PEI-SPION/pDNA) complexes decreased from around 200 nm to 80 nm as the N/P ratio increased. Both PEG-g-PEI-SPION and scAb_GD2_-PEG-g-PEI-SPION condensed pDNA to form stable nanoparticles 80-100 nm in diameter at an N/P ratio of 20. On the other side, the surface charge of the complexes increased with increasing N/P ratio (Figure 2A and 2B).

### Agarose gel retardation assay

The protonated amines groups of PEG-g-PEI-SPION can mediate strong cooperative electrostatic interactions with the negatively charged pDNA, through which pDNA partially or completely loses its negative charge, resulting in the retardation of its migration into the gel. As shown in Figure 2C, PEG-g-PEI-SPION and scAb_GD2_-PEG-g-PEI-SPION completely bound pDNA at N/P ratios of 2.3 and 2.4, respectively. The presence of scAb_GD2_ had no effect on the pDNA condensation capacity of PEG-g-PEI-SPION.

### In vitro cytotoxicity assay and biocompatibility of complexes

An assay of CCK-8 was performed to evaluate the cytotoxicity of targeting and nontargeting complexes in hBMSCs. As shown in Figure 2D, the cytotoxicity of the complexes varied with the N/P ratio in the culture medium. Although higher N/P ratios result in a higher positive surface charge of the nanoparticles, which facilitates the cellular internalization, a higher positive charge is also a major cause of cytotoxicity [26]. At an N/P ratio of 20, the cell viabilities of complexes scAb_GD2_-PEG-g-PEI-SPION/pDNA and PEG-g-PEI-SPION/pDNA were 80.56% ±1.29% and 77.7% ±1.49%, respectively. Furthermore, there were no significant differences between the two groups at different N/P ratios (*P* >0.05), which also demonstrated that the presence of scAb_GD2_ did not increase the cytotoxicity of PEG-g-PEI-SPION.

**Figure 3:**
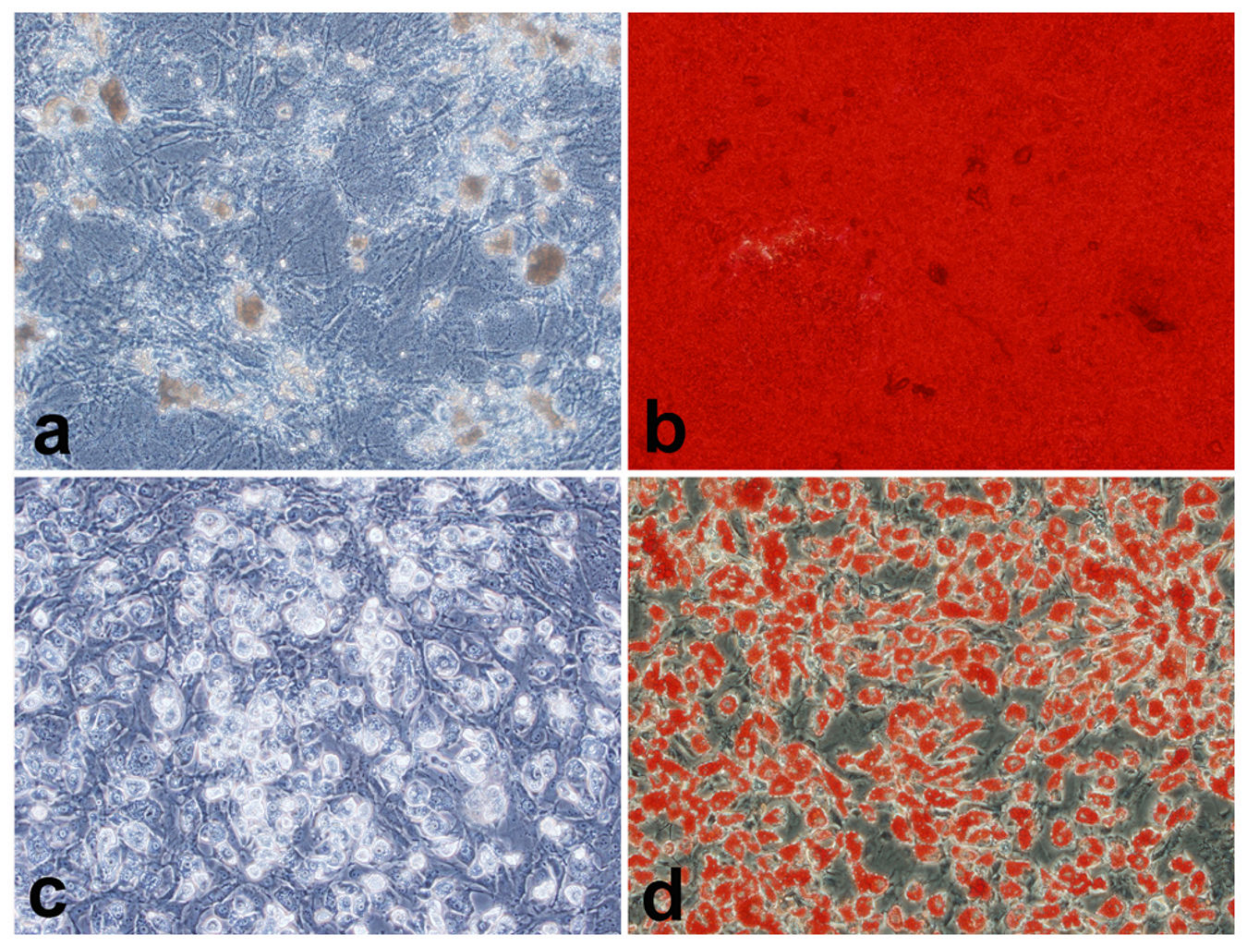
Multilineage potential of hBMSCs after incubation with the complexes. a) unstained osteoblasts, b) stained osteoblasts, c) unstained adipocytes, d) stained adipocytes. After incubation with complexes formed at an N/P ratio of 20 for 12 h, the hBMSCs were successfully induced to differentiate into osteoblasts (a and b) and adipocytes (c and d). Original magnification × 200.

**Figure 4:**
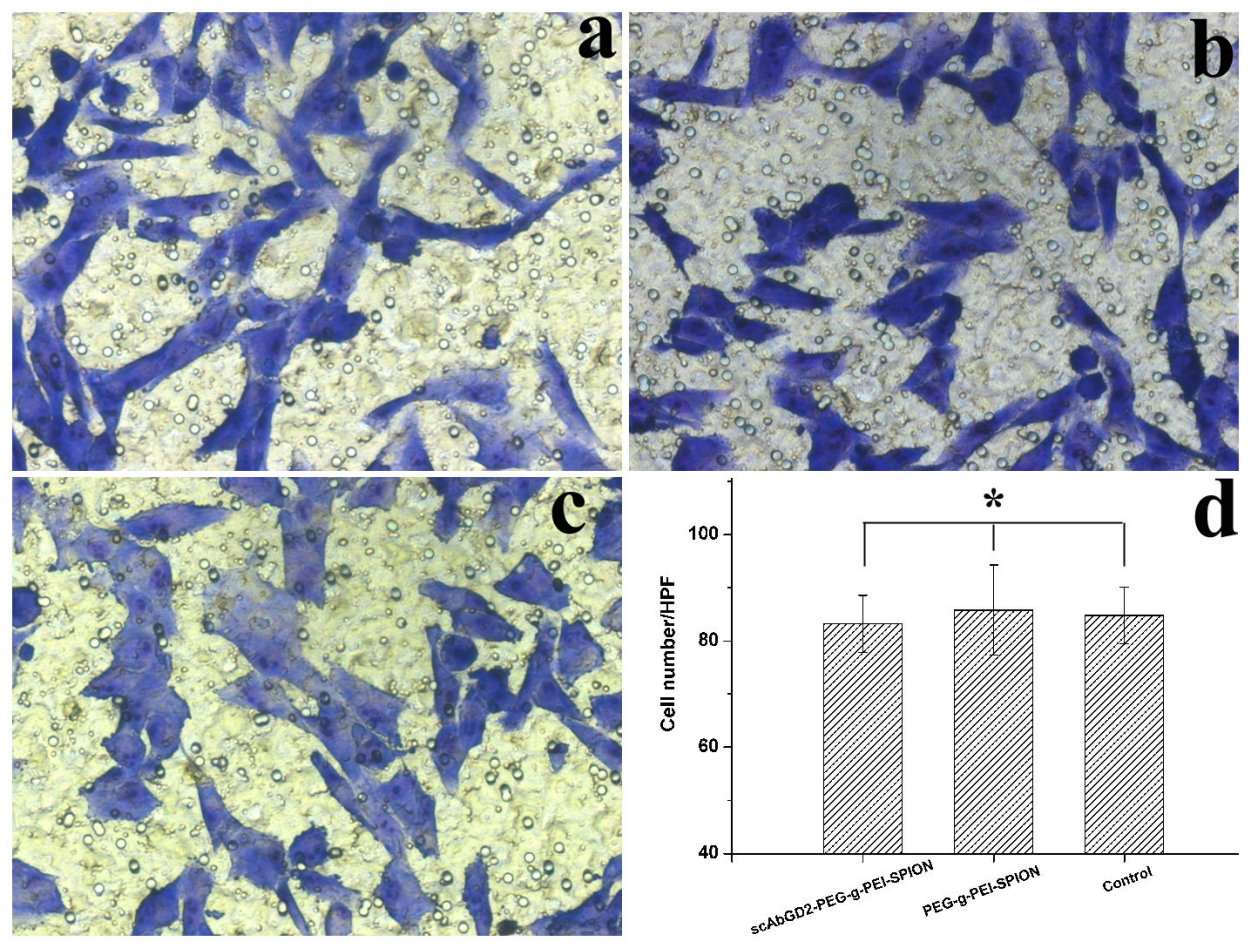
Transwell migration assay. a) hBMSCs incubated with scAb_GD2_-PEG-g-PEI-SPION/pDNA. b) hBMSCs incubated with PEG-g-PEI-SPION/pDNA. c) Normal hBMSCs. hBMSCs penetrated through the permeable membrane were counted in five non-overlapping high power field (d). Complexes scAb_GD2_-PEG-g-PEI-SPION/pDNA and PEG-g-PEI-SPION/pDNA had no effect on the migration ability of hBMSCs as no statistical difference was found among the three groups (*P >0.05). Original magnification × 200.

After being incubated with complexes formed at an N/P ratio of 20 for 12 h, the hBMSCs were successfully induced to differentiate into adipocytes and osteoblasts (Figure 3). Furthermore, we used transwell assay to evaluate the effect of the complexes on the migration ability of hBMSCs. As shown in Figure 4, complexes scAb_GD2_-PEG-g-PEI-SPION/pDNA and PEG-g-PEI-SPION/pDNA had no effect on the migration ability of hBMSCs as no statistical difference was found among the three groups (*P* >0.05). On the whole, the targeting and nontargeting complexes exhibited low cytotoxicity and good biocompatibility at an N/P ratio of 20.

### Gene transfection efficiency

**Figure 5:**
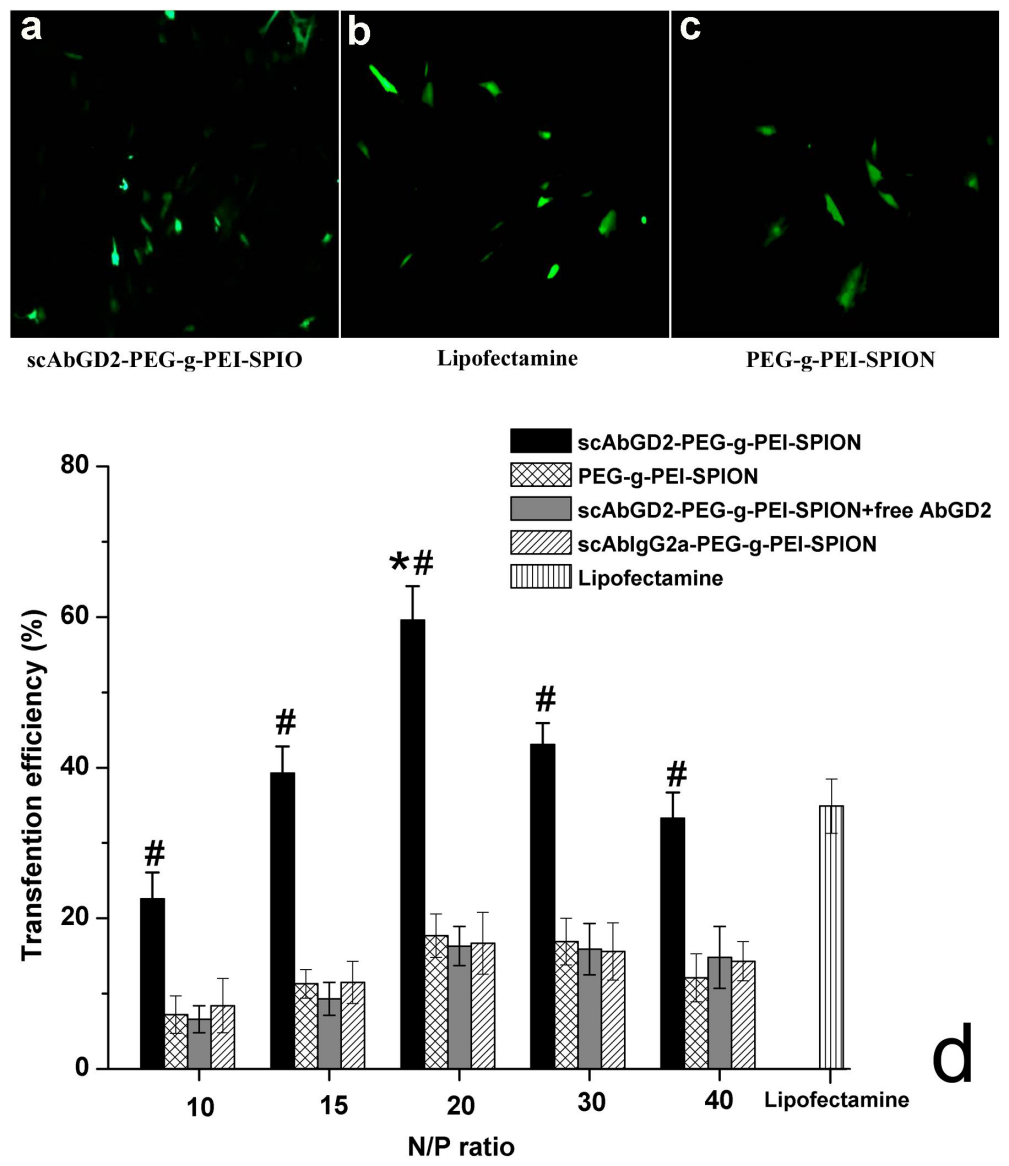
pEGFP-C1 encapsulated by different vectors for gene delivery to hBMSCs. The expression of enhanced green fluorescent protein (EGFP) in hBMSCs transfected with complexes such as scAb_GD2_-PEG-g-PEI-SPION/pDNA (a), lipofectamine/pDNA (b), and PEG-g-PEI-SPION/pDNA (c) was observed using an inverted fluorescence microscope and was quantified by flow cytometry (d). The transfection efficiency of scAb_GD2_-PEG-g-PEI-SPION/pDNA was significantly higher than those of PEG-g-PEI-SPION/pDNA, scAb_GD2_-PEG-g-PEI-SPION/pDNA + free Ab_GD2_, and scAb_IgG2a_-PEG-g-PEI-SPION/pDNA at the same N/P ratio. #*P*<0.01 for scAb_GD2_-PEG-g-PEI-SPION/pDNA versus scAb_GD2_-PEG-g-PEI-SPION/pDNA + free Ab_GD2_, PEG-g-PEI-SPION/pDNA, and scAb_IgG2a_-PEG-g-PEI-SPION/pDNA at the same N/P ratio. *P <0.01 for scAb_GD2_-PEG-g-PEI-SPION/pDNA formed at N/P 20 versus scAb_GD2_-PEG-g-PEI-SPION/pDNA formed at N/P 10, 15, 30, 40, and lipofectamine/pDNA. Original magnification ×100.

hBMSCs incubated with complexes such as scAb_GD2_-PEG-g-PEI-SPION/pDNA, PEG-g-PEI-SPION/pDNA, and scAb_IgG2a_-PEG-g-PEI-SPION/pDNA formed at N/P ratios of 0, 10, 15, 20, 30, and 40 composed the experimental groups. Lipofectamine/pDNA was used as a reference. The expression of EGFP was observed using an inverted fluorescence microscope and was quantified by flow cytometry. As shown in Figure 5, at the same N/P ratio, the transfection efficiency of scAb_GD2_-PEG-g-PEI-SPION/pDNA was significantly higher than those of PEG-g-PEI-SPION/pDNA, scAb_GD2_-PEG-g-PEI-SPION/pDNA + free Ab_GD2_, and scAb_IgG2a_-PEG-g-PEI-SPION/pDNA (*P*<0.01). At the optimal N/P ratio of 20, the transfection efficiency of complex scAb_GD2_-PEG-g-PEI-SPION/pDNA reached the highest value at 59.6% ± 4.5%, while PEG-g-PEI-SPION/pDNA, scAb_GD2_-PEG-g-PEI-SPION/pDNA + free Ab_GD2_, and scAb_IgG2a_-PEG-g-PEI-SPION/pDNA reached their highest transfection efficiencies at 17.7% ± 2.9%, 16.3% ± 2.6%, and 16.7% ± 4.1%, respectively. The transfection efficiency using complex lipofectamine/pDNA was 34.9% ± 3.6%. The cells incubated with naked pEGFP (N/P ratio of 0) exhibited no fluorescence (data not shown). So far, viral vectors are mostly used to deliver genes to stem cells [13,14]. Although satisfactory transfection efficiency has been achieved, their safety risks such as immunogenicity restrict further application of them to humans. Non-viral gene vectors such as PEI posses the superiority of stability, safety, ease in preparation, and lack of immunogenicity. However, compared with viral vectors, the efficiency of non-viral vectors for gene delivery to stem cells is relatively low [20,27]. In this study, the highest transfection efficiency of complex PEG-g-PEI-SPION/pDNA was only 17.7% ± 2.9%. We successfully constructed scAb_GD2_-PEG-g-PEI-SPION as a targeted and efficient non-viral vector for gene delivery to hBMSCs, and significantly enhanced transgene expression in hBMSCs was achieved via the GD2 receptor-mediated endocytosis of the complexes.

### Cellular uptake of complexes and Prussian blue staining

**Figure 6:**
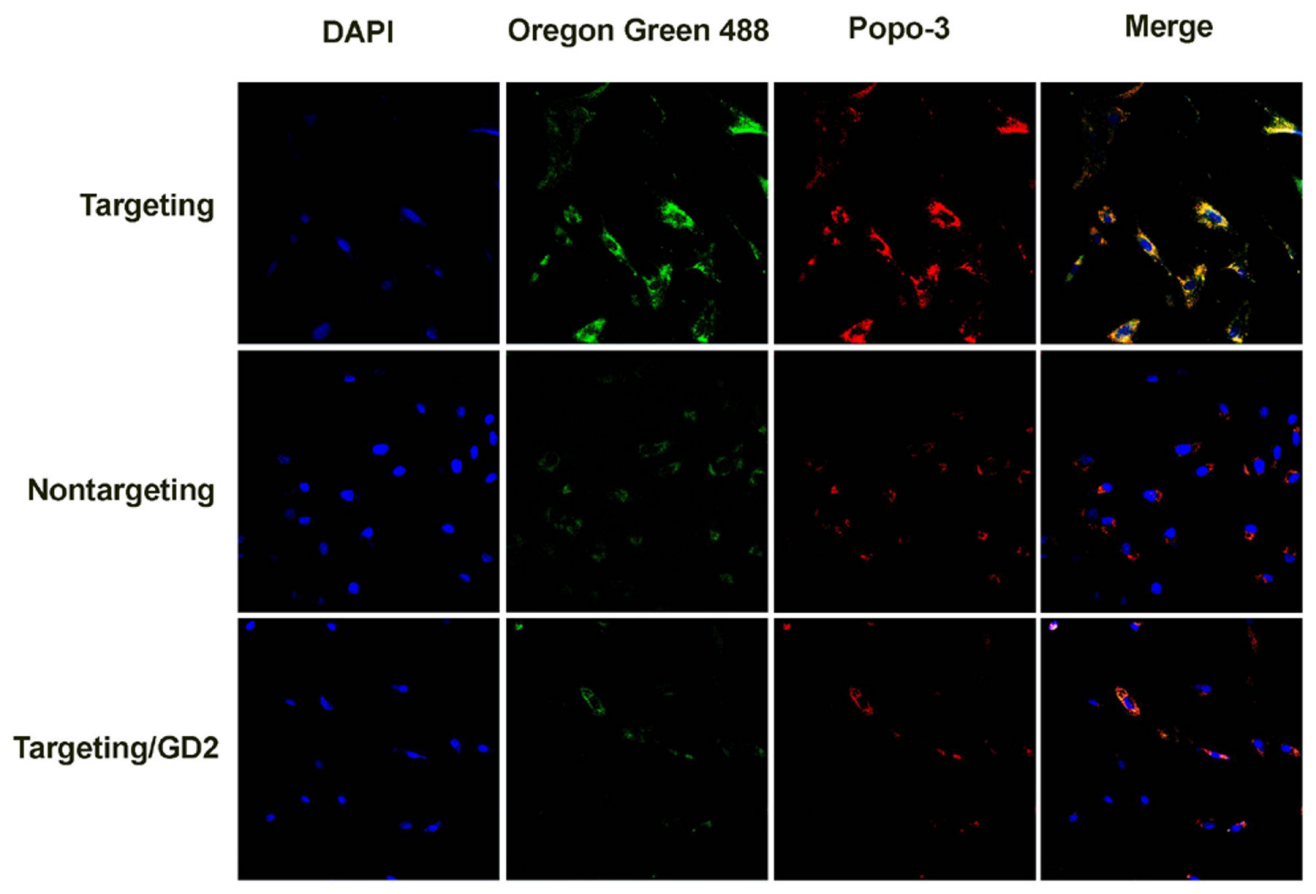
Cellular internalization of the complexes. Images assignment: green: delivery agent, blue: nucleus, Red: pDNA. The image on the right is an overlay of the three fluorescent colors. Cells incubated with targeting complex displayed clearly stronger delivery agent (green) and pDNA (red) fluorescence compared with cells incubated with nontargeting and targeting/GD2 complexes.

**Figure 7:**
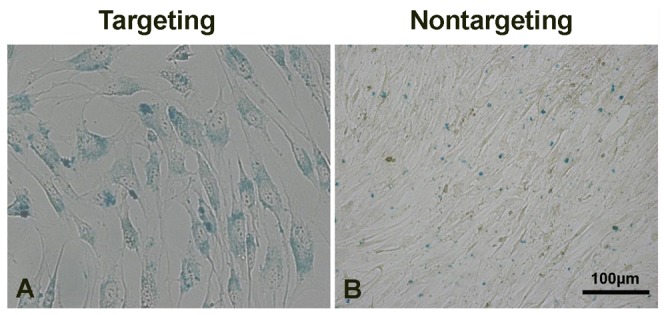
Prussian blue staining. Compared with the cells incubated with nontargeting complex (B), cells incubated with targeting complex displayed much more intensive blue color (A), indicating much more intracellular SPION.

The cellular uptake of the complexes was visualized by the results of CLSM experiment. Notably, cells incubated with scAb_GD2_-PEG-*g*-PEI-SPION/pDNA (targeting complex) displayed clearly stronger delivery agent (green) and pDNA (red) fluorescence compared with cells incubated with PEG-*g*-PEI-SPION/pDNA (nontargeting complex) and scAb_GD2_-PEG-g-PEI-SPION/pDNA + free Ab_GD2_ (targeting/GD2) (Figure 6). Prussian blue staining was performed to confirm the presence of SPION in the cytoplasm of the cells. Compared with the cells incubated with nontargeting complex, cells incubated with targeting complex displayed much more intensive blue color, indicating much more intracellular SPION (Figure 7). These results also evidenced that enhanced cellular uptake of targeting complex was mediated by the specific interaction between the scAb_GD2_ and GD2 receptors.

### In vitro MRI scan

**Figure 8:**
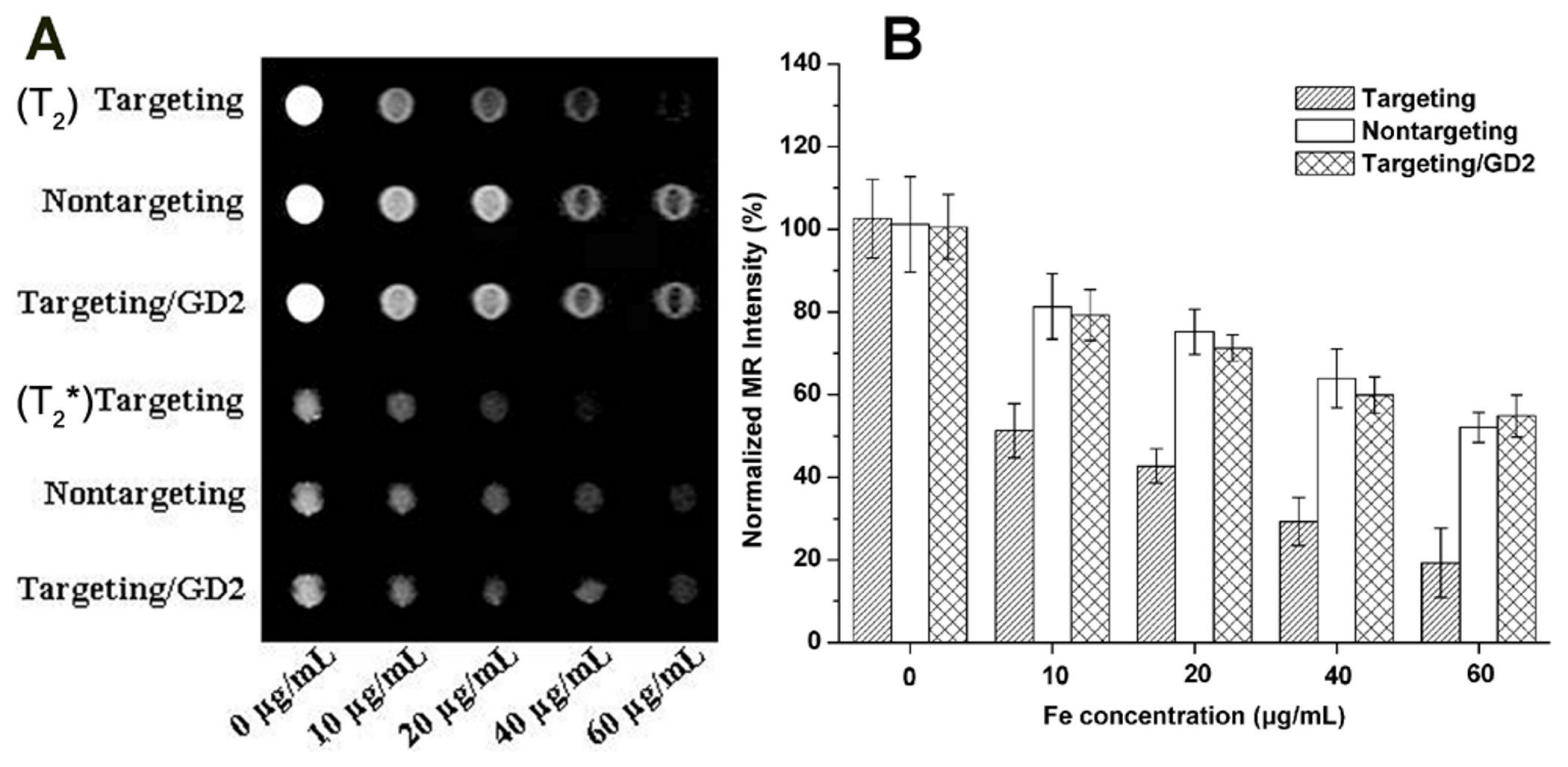
*In vitro* MRI scan. The cells were scanned with a 1.5 T MRI scanner at room temperature (A). All complexes were formed at an N/P ratio of 20. B shows the normalized MR signal intensity of different complexes at various Fe concentrations (n=3).

The non-viral vector was visible on MRI by forming nanoparticulate complexed with SPION which was a highly efficient contrast agent for T_2_/T_2_*-weighted MR images. As shown in Figure 8, hBMSCs exhibited low signal intensity on T_2_/T_2_*-weighted images due to the presence of SPION in the cytoplasm of the cells. The MRI signal intensity of cells incubated with complexes decreased with the increase of the Fe concentration. At the same Fe concentration, cells incubated with targeting complex displayed a significant decrease compared with when they were incubated with nontargeting and targeting/GD2 complexes, which was consistent with the results of CLSM and Prussian blue staining experiments.

### In vivo gene expression

**Figure 9:**
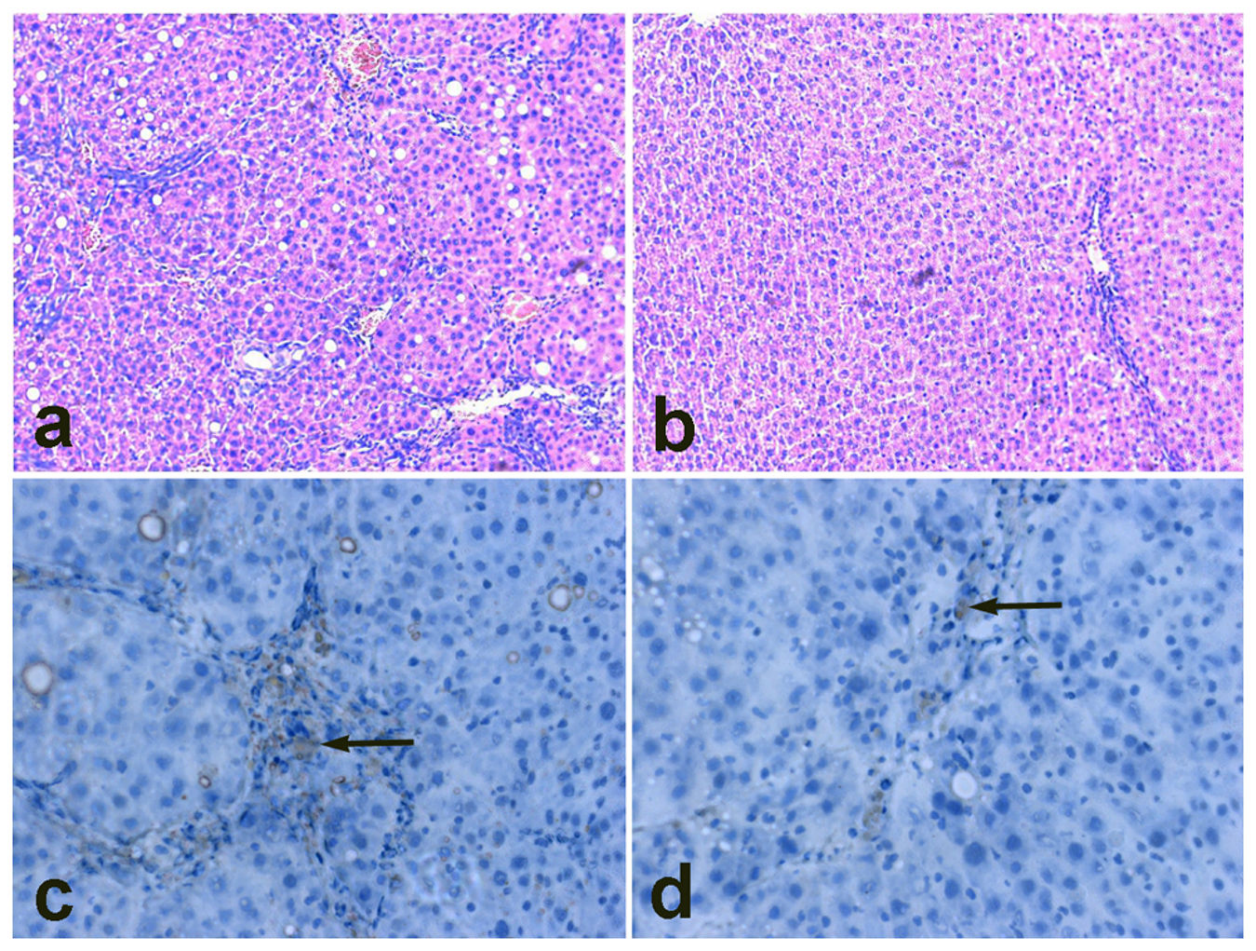
Micrographs of histological specimens of liver tissue (×200). Liver fibrosis was successfully induced by CCL_4_ after 10 weeks. Hematoxylin-eosin staining shows fibrotic (a) and normal (b) rat livers. Compared with the nontargeting group (d), more EGFP-positive cells were observed in the targeting group (c).

**Figure 10:**
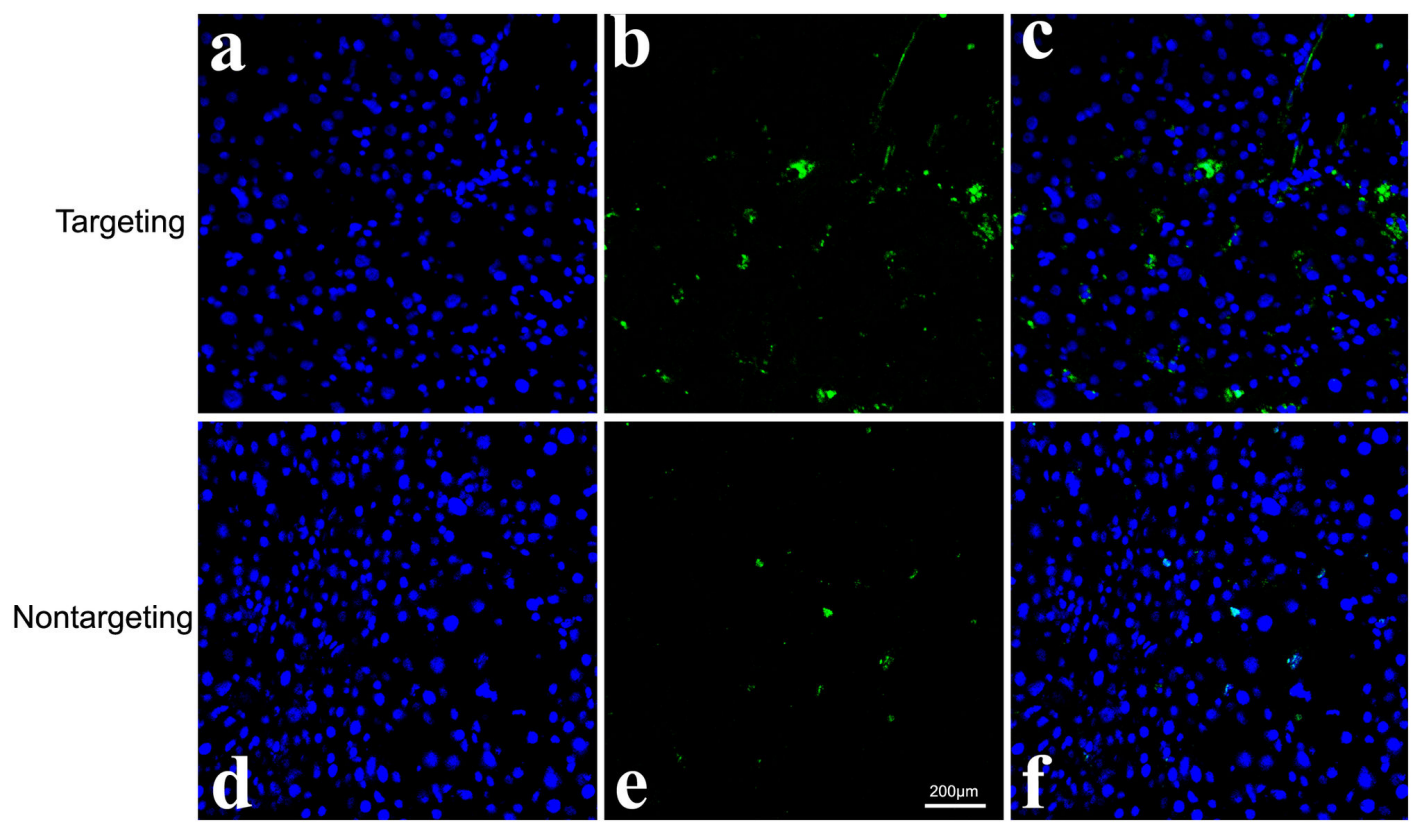
Confocal laser scanning microscopic images of frozen sections. a, b, c) targeting group; d, e,f) nontargeting group. Images assignment: green: EGFP fluorescence, blue: nucleus. The image on the right is an overlay of the two fluorescent colors. Confocal laser scanning microscopic images of frozen sections showed that more EGFP fluorescence was observed in the targeting group.

Liver fibrosis was successfully induced by CCL_4_ after 10 weeks. EGFP-positive cells were found in the portal triad region at the third day after transplantation (Figure 9). Furthermore, compared with the nontargeting group, more EGFP-positive cells and EGFP fluorescence were observed in the targeting group (Figure 9 and Figure 10). Labeling and tracking of stem cells are crucial for assessing cell distribution and homing. MRI is the preferred noninvasive imaging modality for tracking of transplanted stem cells labeled with magnetically visible contrast agents in hepatic diseases. In this study, we have initially proved that the complex scAb_GD2_-PEG-g-PEI-SPION/pDNA has better targeting tropism to GD2-positive hBMSCs than its nontargeting counterparts, and MRI has the potential to monitor this targeting event in a noninvasive way. We are currently conducting animal tests to track the transplanted GD2-positive hBMSCs in fibrotic rat livers by MRI. Theoretically, after internalization of the targeting complex, the transfected hBMSCs retained in the fibrotic rat livers would express a reporter/therapeutic gene and display low signal intensity on T_2_*-weighted images.

## Conclusion

In summary, we constructed an MRI-visible non-viral gene delivery vector bearing GD2 single chain antibody (scAb_GD2_) as a targeting ligand to hBMSCs. At an optimal N/P ratio for plasmid delivery, the targeting scAb_GD2_-PEG-g-PEI-SPION/pDNA nanoparticles showed low cytotoxicity, good biocompatibility, and sensitive signal on T_2_/ T_2_*-weighted MR images in vitro. Our study demonstrated that the complex scAb_GD2_-PEG-g-PEI-SPION/pDNA had better targeting tropism to GD2-positive hBMSCs than its nontargeting counterparts and the reporter genes (EGFP) were highly expressed in vitro and in vivo.
